# Insidious Mass Within a Sinus

**DOI:** 10.14797/mdcvj.1316

**Published:** 2023-12-29

**Authors:** Aneesh Dhorepatil, Vivek Modi, Mahwash Kassi, Mohammad A. R. Chamsi-Pasha, April Ewton, Dyron Allen, Mouaz H. Al Mallah

**Affiliations:** 1 Houston Methodist DeBakey Heart & Vascular Center, Houston, Texas, US; 2 Houston Methodist Hospital, Houston, Texas, US

**Keywords:** cardiac MRI, cardiac PET, histo-pathology

## Abstract

A 75-year-old patient was incidentally found to have an intracardiac mass by echocardiography. Subsequent cardiac magnetic resonance imaging and cardiac positron emission tomography confirmed a large and possibly malignant mass extending from the right atrium into the coronary sinus.

The patient underwent an intracardiac echocardiography guided biopsy, which revealed diffuse B-cell lymphoma, and is currently undergoing rituximab, etoposide, vincristine, cyclophosphamide, and doxorubicin (R-EPOCH)-based chemotherapy.

## Brief Description

A 75-year-old patient with a past medical history of coronary artery disease, peripheral vascular disease, diabetes mellitus, and hypertension was incidentally found to have an intracardiac mass by echocardiography performed at an outside institution.

**Figure 1:**
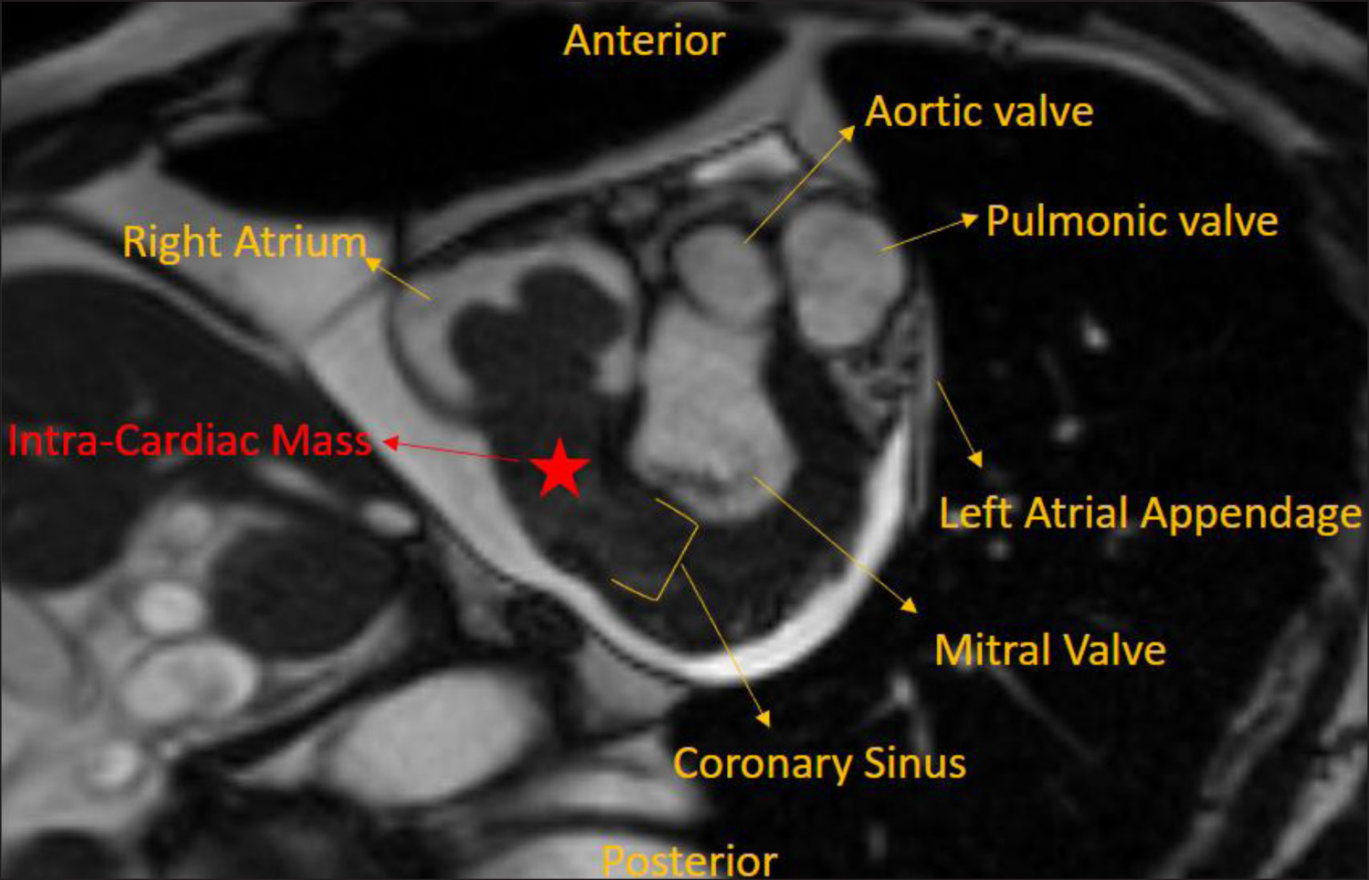
Left atrial view showing the large mass in the right atrium extending into the coronary sinus.

**Figure 2:**
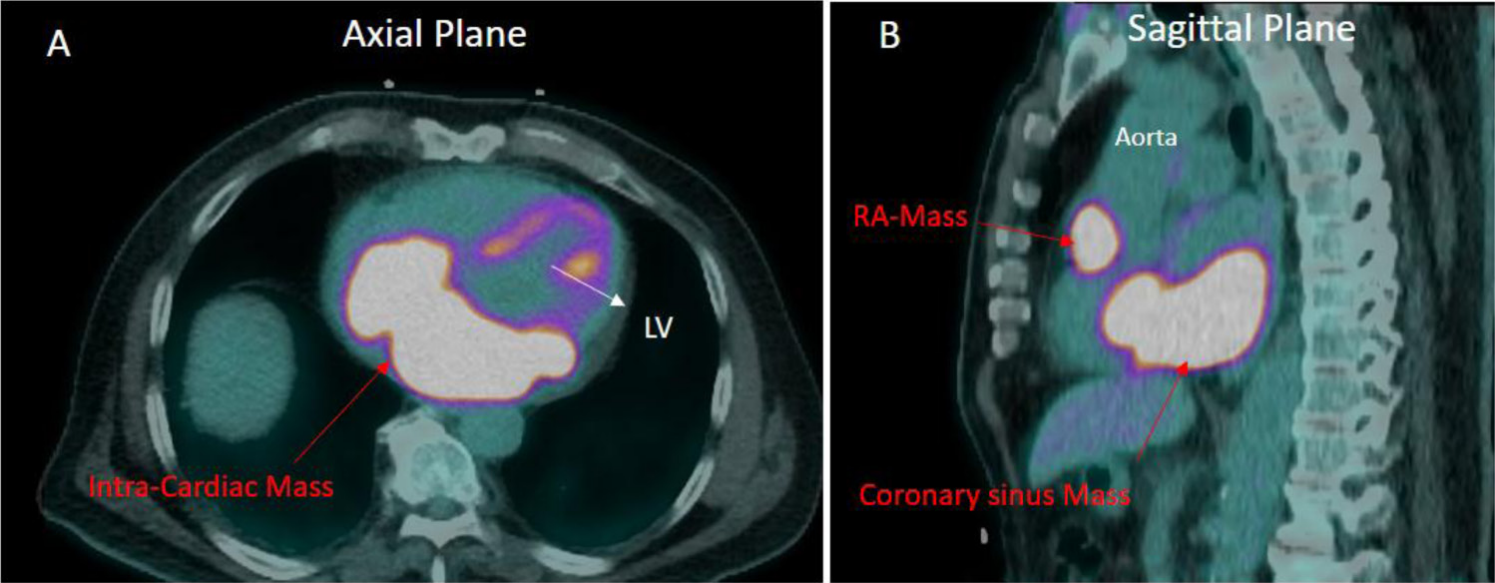
**(A)** Axial plane image demonstrating intense fluorodeoxyglucose (FDG) uptake in the intracardiac mass (SUV Max 18). **(B)** Sagittal plane image demonstrating a second intracardiac mass in the wall of the right atrium, which also has intense FDG uptake (SUV Max 12). Intense FDG uptake in both masses is consistent with a malignant tumor. LV: left ventricle; RA: right atrium

**Figure 3:**
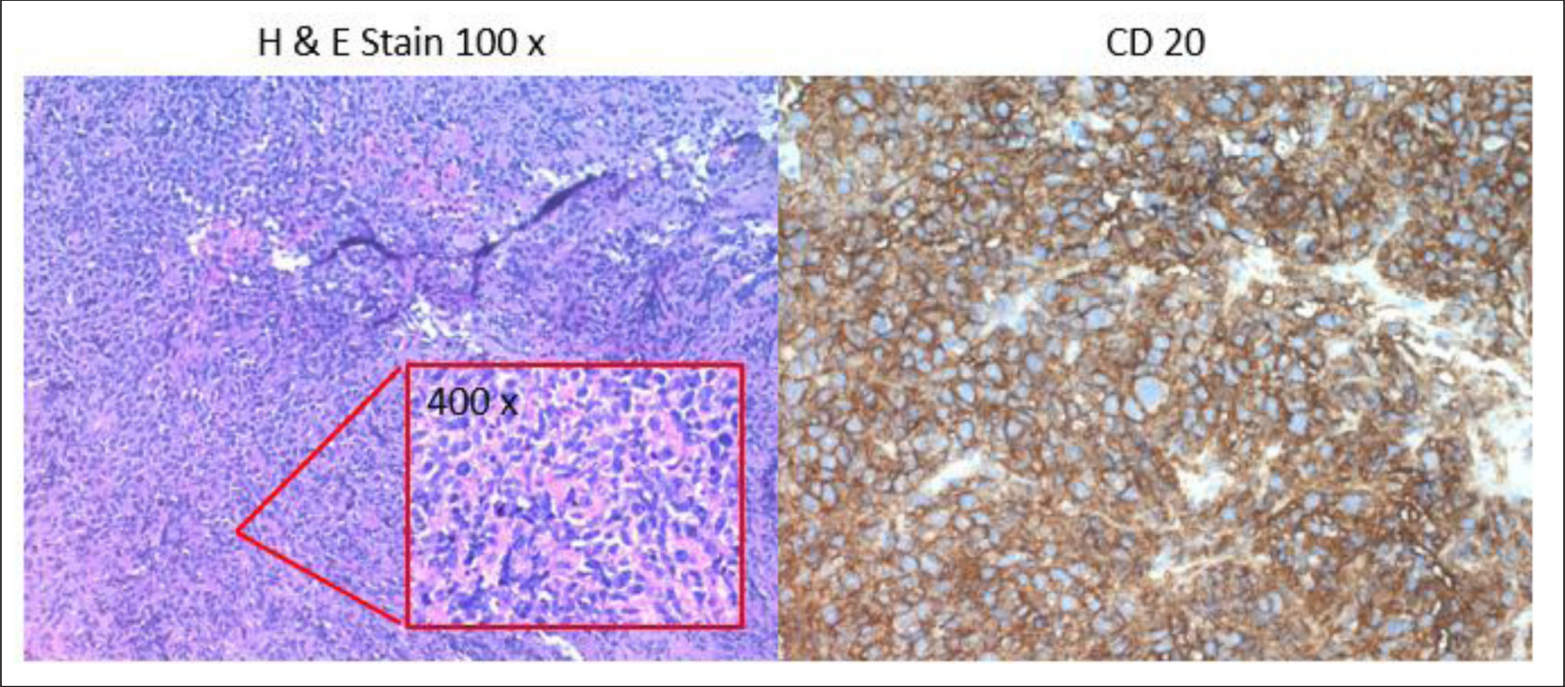
Histologic sections of the atrial mass biopsy showed a diffuse infiltrate of large atypical lymphocytes with areas of necrosis. Immunohistochemical stains showed that the lymphocytes were positive for CD20, BCL6, and MUM-1 and negative for CD10. FISH studies were negative for rearrangements of MYC, BCL2, and BCL6. The overall findings were those of diffuse large B-cell lymphoma, non-germinal center immunophenotype. FISH: fluorescence in situ hybridization

Subsequently, the patient underwent cardiac magnetic resonance imaging to confirm the presence and extent of the mass (Figure 1; Video 1). A cardiac positron emission tomography study was performed in order to determine if the mass was a benign or malignant tumor (Figure 2). The patient underwent an intracardiac echocardiography guided biopsy, which revealed diffuse B-cell lymphoma (Figure 3).

**Video 1:** Axial plane cardiac magnetic resonance image demonstrating the extent of the intracardiac mass, which starts in the right atrium and extends into the coronary sinus. Incidentally, the patient has a large pericardial effusion without tamponade physiology. See also at https://youtu.be/y9w65OyMKNs.

As the lymphoma is chemo sensitive, the patient is currently undergoing rituximab, etoposide, vincristine, cyclophosphamide, and doxorubicin (R-EPOCH) based chemotherapy.

